# Pulmonary embolism or thrombosis in ARDS COVID-19 patients: A French monocenter retrospective study

**DOI:** 10.1371/journal.pone.0238413

**Published:** 2020-08-27

**Authors:** Damien Contou, Olivier Pajot, Radj Cally, Elsa Logre, Megan Fraissé, Hervé Mentec, Gaëtan Plantefève

**Affiliations:** Service de réanimation polyvalente, Centre Hospitalier Victor Dupouy, Argenteuil, France; Institut d’Investigacions Biomediques de Barcelona, SPAIN

## Abstract

Hypercoagulability and endotheliopathy reported in patients with coronavirus disease 2019 (COVID-19) combined with strict and prolonged immobilization inherent to deep sedation and administration of neuromuscular blockers for Acute Respiratory Distress Syndrome (ARDS) may expose critically ill COVID-19 patients to an increased risk of venous thrombosis and pulmonary embolism (PE). We aimed to assess the rate and to describe the clinical features and the outcomes of ARDS COVID-19 patients diagnosed with PE during ICU stay. From March 13^th^ to April 24^th^ 2020, a total of 92 patients (median age: 61 years, 1st-3rd quartiles [55–70]; males: n = 73/92, 79%; baseline SOFA: 4 [3–7] and SAPS II: 31 [21–40]; invasive mechanical ventilation: n = 83/92, 90%; ICU mortality: n = 45/92, 49%) were admitted to our 41-bed COVID-19 ICU for ARDS due to COVID-19. Among them, 26 patients (n = 26/92, 28%) underwent a Computed Tomography Pulmonary Angiography which revealed PE in 16 (n = 16/26, 62%) of them, accounting for 17% (n = 16/92) of the whole cohort. PE was bilateral in 3 (19%) patients and unilateral in 13 (81%) patients. The most proximal thrombus was localized in main (n = 4, 25%), lobar (n = 2, 12%) or segmental (n = 10, 63%) pulmonary artery. Most of the thrombi (n = 13/16, 81%) were located in a parenchymatous condensation. Only three of the 16 patients (19%) had lower limb venous thrombosis on Doppler ultrasound. Three patients were treated with alteplase and anticoagulation (n = 3/16, 19%) while the 13 others (n = 13/16, 81%) were treated with anticoagulation alone. ICU mortality was higher in patients with PE compared to that of patients without PE (n = 11/16, 69% vs. n = 2/10, 20%; p = 0.04). The low rate of lower limb venous thrombosis together with the high rate of distal pulmonary thrombus argue for a local immuno-thrombotic process associated with the classic embolic process. Further larger studies are needed to assess the real prevalence and the risk factors of pulmonary embolism/thrombosis together with its prognostic impact on critically ill patients with COVID-19.

## Introduction

Severe acute respiratory syndrome coronavirus-2 (SARS-CoV-2) is the novel coronavirus originating from Wuhan, China, responsible for the illness named Coronavirus disease 2019 (COVID-19) that has rapidly spread worldwide especially in Europe. Chinese [1–3] and American [4, 5] reports on critically ill patients with COVID-19 describe a poor outcome with high mortality rate, especially in patients requiring invasive mechanical ventilation. Hypercoagulability [6, 7] and endotheliopathy [8–10] reported in patients with COVID-19 combined with strict and prolonged immobilization inherent to deep sedation and administration of neuromuscular blockers for Acute Respiratory Distress Syndrome (ARDS) may expose critically ill COVID-19 patients to an increased risk of venous thrombosis and pulmonary embolism [11–13] (PE). We aimed to assess the rate and to describe the clinical features and the outcomes of ARDS COVID-19 patients diagnosed with PE during ICU stay.

## Patients and methods

All Computed Tomography Pulmonary Angiographies (CTPA) performed in critically ill adult COVID-19 patients (RT-PCR positive for SARS-CoV-2) because of sudden circulatory (introduction or significant increase of the dose of vasopressor) or/and respiratory (significant increase of FiO_2_ requirement) worsening—with no obvious explanation such as ventilatory associated pneumonia or other source of sepsis—were retrospectively reviewed. The patient had to be safely transportable and the final decision to perform a CTPA was left at the discretion of the treating intensivist in charge. Clinical data and laboratory results were collected the day of CTPA. All the patients diagnosed with PE had venous Doppler ultrasound of the lower limbs.

Continuous variables were reported in median [interquartile range] and were compared between groups using the Mann-Whitney test. Categorical variables were reported in numbers (percentage) and compared using Fisher’s exact test. A p value < 0.05 was considered statistically significant. The statistical analysis was performed by using the RStudio software version 0.99.441 (www.rStudio.com).

This observational, non-interventional analysis of medical records of patients hospitalized in Argenteuil ICU (France) from March 13^th^ to April 24^th^ 2020, was conducted in accordance with the amended Declaration of Helsinki and was approved (CE 20–39) by the Institutional Review Board of the French Intensive Care Society (FICS). All the data were fully anonymized. As per French law, no informed consent was required for this type of study.

## Results

From March 13^th^ to April 24^th^ 2020, a total of 92 patients were admitted to our 41-bed COVID-19 ICU (Argenteuil, France) for ARDS due to SARS-CoV-2 pneumonia. The main characteristics, comorbidities, biological data and outcomes of the 92 COVID-19 patients admitted to our ICU are detailed in the Table 1.

**Table 1:** Main characteristics, comorbidities, biological data and outcomes of the 92 COVID-19 patients admitted to our ICU.

	Critically ill patients with SARS-CoV-2 pneumonia n = 92
Age, years	61 [55–70]
Male, n (%)	73 (79)
Baseline SOFA	4 [3–7]
Baseline SAPS II	31 [21–40]
**Main comorbidities, n (%)**	
Obesity (body mass index ≥ 30 kg/m^2^)	38 (41)
Hypertension	59 (64)
Diabetes mellitus	35 (38)
Cardio-vascular diseases	9 (10)
Atrial fibrillation	3 (3)
Cerebro-vascular diseases	8 (9)
Venous thrombo-embolism	5 (5)
Chronic respiratory diseases^a^	18 (20)
Chronic renal failure	7 (8)
Immunocompromised status^b^	9 (10)
**Biological data at ICU admission**	
Leukocytes count, 10^3^/mm^3^	9.0 [6.8–12.2]
Lymphocytes count, 10^3^/mm^3^	0.8 [0.6–1.1]
Platelets count, 10^3^/mm^3^	226 [183–303]
C-reactive protein, mg/L	175 [131–232]
Procalcitonin, ng/mL	0.9 [0.3–2.2]
Fibrinogen, g/L	7.7 [6.1–8.8]
**Outcomes in ICU**	
Invasive mechanical ventilation	83 (90)
Prone positioning	55 (60)
Vasopressor support	57 (62)
Renal replacement therapy	22 (24)
ICU mortality	45 (49)

^a^including Chronic Obstructive Pulmonary Disease (n = 6) or/and obstructive sleep apnea (n = 12) or/and asthma (n = 4).

^b^including chronic lymphocytic leukemia (n = 2), follicular or Hodgkin lymphoma (n = 2), liver transplantation (n = 1), long term corticosteroid therapy (>0.5mg/kg for more than 3 months) (n = 3) or azathioprine (n = 1) administration.

Continuous variables are reported as median [Interquartile range] and categorical variables are reported as numbers (percentage).

Table abbreviations

ICU: Intensive Care Unit; SOFA: Sequential Organ Failure Assessment; SAPS II: Simplified Acute Physiology Score II.

At the time of analysis (April 24^th^), 26 patients (n = 26/92, 28%) underwent a CTPA which revealed PE in 16 (n = 16/26, 62%) of them, accounting for 17% (n = 16/92) of the whole cohort. Clinical features, outcomes and comparison between ARDS COVID-19 patients with (n = 16) and without (n = 10) PE are detailed in the Table 2. D-dimer (5.3 vs. 1.9 μg/mL, p = 0.32), fibrinogen (7.8 vs. 7.8 g/L, p = 0.49) and platelet count (347 vs. 349 10^3^/mm^3^, p = 0.64) did not significantly differ between patients with and without PE (Table 2). Compared to patients diagnosed with PE, the number of days between ICU admission and CTPA was higher in patients without PE (7 vs. 17 days, p = 0.008) (Table 2).

**Table 2:** Comparison of ARDS COVID-19 patients with (n = 16) or without (n = 10) pulmonary embolism/thrombosis.

	COVID-19 ARDS patients with PE N = 16	COVID-19 ARDS patients without PE N = 10	p
**Patients characteristics and ICU scores**			
Male sex, n (%)	14 (89%)	8 (80%)	0.63
Age, years	63 [47–77]	63 [46–73]	0.89
Body mass index, kg/m^2^	29 [19–43]	28 [25–43]	0.60
Baseline SOFA	4 [2–18]	4.5 [2–11]	0.83
Baseline SAPS II	33 [16–88]	31 [16–56]	0.73
**Comorbidities, n (%)**			
Diabetes mellitus	6 (38%)	4 (40%)	1
Hypertension	9 (56%)	6 (60%)	1
Obesity (Body Mass Index>30 kg/m^2^)	7 (44%)	3 (30%)	0.68
Ischemic cardiopathy	1 (6%)	1 (10%)	1
Recent cancer or malignant hemopathy	0 (0%)	0 (0%)	1
Previous venous thrombo-embolic disease	0 (0%)	0 (0%)	1
None	4 (25%)	1 (10%)	0.62
**Main delays**			
Days between disease onset and ICU admission	7 [3–10]	8 [4–11]	0.33
Days between ICU admission and CTPA	7 [1–24]	17 [8–35]	0.008
Days between disease onset and CTPA	15.5 [8–29]	25 [14–42]	0.008
**Laboratory data the day of CTPA**			
D-dimer, μg/mL	5.3 [1.8–20]	1.9 [0.5–19]	0.32
Fibrinogen, g/L	7.8 [3.2–11.7]	7.8 [4.1–9]	0.49
Platelet count, 10^3^/mm^3^	347 [50–558]	349 [142–437]	0.64
**Thromboprophylaxis, n (%)**	16 (100%)	10 (100%)	
Calcium heparin	9 (56%)	3 (30%)	0.25
Sodium heparin	1 (6%)	1 (10%)	1
Fondaparinux	3 (19%)	3 (30%)	0.64
Enoxaparin	3 (19%)	3 (30%)	0.64
Leg compression	0 (0%)	0 (0%)	1
**Reason(s) for performing CTPA, n (%)**			
Circulatory worsening	3 (19%)	1 (10%)	1
Respiratory worsening	8 (50%)	9 (90%)	0.08
Circulatory and respiratory worsening	5 (31%)	0 (0%)	0.12
**ARDS according to Berlin definition**	**16 (100%)**	**10 (100%)**	-
Severe, n (%)	7 (44%)	3 (30%)	0.68
Moderate, n (%)	9 (56%)	5 (50%)	1
Mild, n (%)	0 (0%)	2 (20%)	0.14
PaO_2_/FiO_2_ ratio, mmHg	109 [64–188]	142 [72–265]	0.24
Prone positioning, n (%)	10 (63%)	9 (90%)	0.19
Neuromuscular blockers, n (%)	16 (100%)	10 (100%)	1
**Circulatory and kidney failures**			
Norepinephrine, n (%)	7 (44%)	1 (10%)	0.09
Median dose of norepinephrine, μg/kg/min	0.58 [0.10–1.50]	0.2 [0.2–0.2]	-
Acute cor pulmonar, n (%)	4 (25%)	0 (0%)	0.14
*De novo* atrial fibrillation, n (%)	6 (38%)	1 (10%)	0.19
Renal replacement therapy for acute kidney failure, n (%)	7 (44%)	4 (40%)	1
**Outcome**			
Death in ICU, n (%)	11 (69%)	2 (20%)	0.04
Still under invasive ventilation, n (%)	0 (0%)	1 (10%)	0.38
Discharged to the wards, n (%)	5 (31%)	7 (70%)	0.10

Table abbreviations

ARDS: Acute Respiratory Disease Syndrome; BMI: Body Mass Index; COVID-19: Coronavirus disease 2019; CTPA: Computed Tomography Pulmonary Angiographies; ICU: Intensive Care Unit; HIV: Human Immunodeficiency Virus; PE: Pulmonary Embolism; SOFA: Sequential Organ Failure Assessment; SAPS II: Simplified Acute Physiology Score II.

Continuous variables are reported as median [Interquartile range] and categorical variables are reported as numbers (percentage).

**Fig 1:**
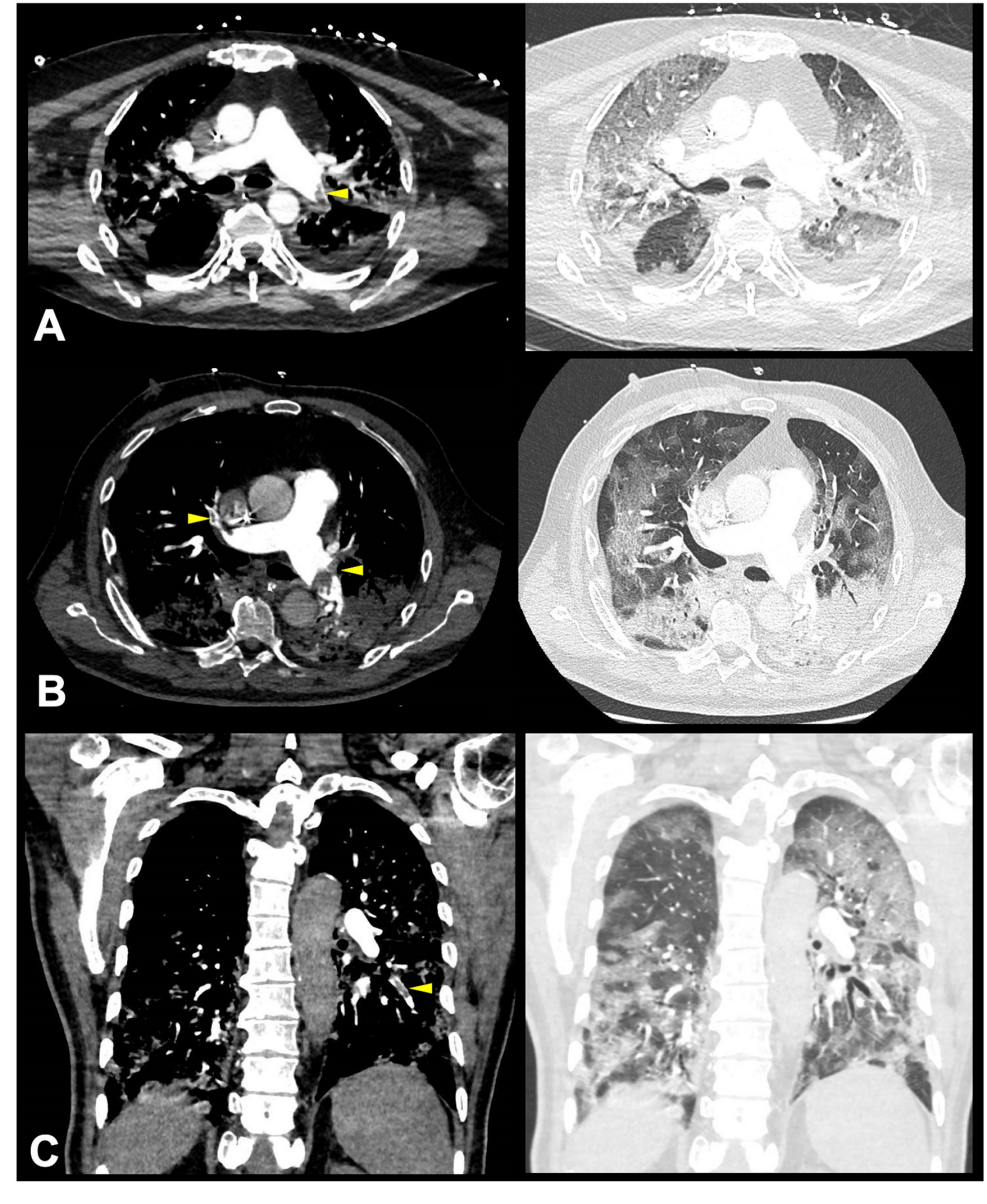
Pulmonary embolism or thrombosis in ARDS COVID-19 patients. Illustrative examples of Computed Tomography Pulmonary Angiography revealing intraluminal defects (left side, yellow arrowhead) and associated parenchymatous condensations (right side) in the left lower pulmonary artery in a 73-year-old patient at day 5 after ICU admission (Panel A), in the main left pulmonary artery and in the anterior segmental pulmonary artery of the right upper lobe in a 73-year-old patient at day 4 after ICU admission (Panel B) and in a segmental pulmonary artery of the left lower lobe (coronal view) in a 65-year-old patient at day 3 after ICU admission (Panel C).

PE was bilateral in 3 (19%) patients and unilateral in 13 (81%) patients. The most proximal thrombus was localized in main (n = 4, 25%), lobar (n = 2, 12%) or segmental (n = 10, 63%) pulmonary artery. Most of the thrombi (n = 13/16, 81%) were located in a parenchymatous condensation. Illustrative examples are provided in the Fig 1. Only three of the 16 patients (19%) had lower limb venous thrombosis on Doppler ultrasound. Three patients were treated with alteplase and anticoagulation (n = 13/16, 19%) while the 13 others (n = 13/16, 81%) were treated with anticoagulation alone. ICU mortality was higher in patients with PE compared to that of patients without PE (n = 11/16, 69% vs. n = 2/10, 20%; p = 0.04).

## Discussion

We herein report on a 62% rate of PE in critically ill patients with COVID-19 related ARDS in whom a CTPA was performed for respiratory or circulatory worsening, accounting for 17% of the whole cohort of ARDS COVID-19 patients. This 17% rate observed in our cohort is consistent with the 21% and 17% rates recently reported by Poissy (10) and Helms (9), respectively. However, this rate might be underestimated since 24 patients of our whole cohort were still hospitalized in the ICU at the time of analysis. Moreover, CTPAs were not performed systematically but only in case of respiratory and/or circulatory worsening, potentially responsible for an underestimation of the real rate of PE in ARDS COVID-19 patients. Interestingly, the delay between ICU admission and CTPA was significantly shorter in patients diagnosed with PE as compared to those without PE. Hence, PE might occur earlier in the course of COVID-19 ARDS, as compared to other complications of ARDS. Moreover, our delay of 7 days between ICU admission and CTPA (i.e. diagnostic of PE) is in line with the delay of 5.5 days recently reported by Helms et al. [11] in 25 critically ill COVID-19 patients. Whether these PE were acquired during the first seven days of ICU stay or already present at ICU admission (i.e. fortuitus finding not explaining the respiratory or circulatory worsening of the patients) is unknown. Only systematic realization of contrast-enhanced chest CT-scan at ICU admission of COVID-19 patients could answer this question [14].

We report on a significantly higher ICU mortality in patients with PE compared to that of patients without PE. However, no conclusion can be drawn regarding the impact of PE on the outcome of critically ill patients with COVID-19 since CTPAs were performed significantly later in patients without PE exposing them to a potential survival bias.

Despite systematic prophylactic anticoagulant therapy, a clinicopathologic case series found a high rate of thrombosis in small and mid-sized pulmonary arteries in COVID-19 patients [15]. The low rate of lower limb venous thrombosis (usually found in 60% of patients with PE [16]) together with the high rate of distal pulmonary thrombus (mostly located in a parenchymatous condensation) observed in our patients question about a local immuno-thrombotic process (pulmonary vascular endothelialitis [9, 10]) associated with the classic embolic process.

The retrospective monocenter design of our study implies numerous limitations. Nevertheless, intensivists should be aware of a possible increased risk of PE in critically ill COVID-19 patients with ARDS, despite systematic prescription of thromboprophylaxis. Our data suggest that CTPA should be easily performed–even when renal function is impaired–in case of sudden respiratory and/or circulatory worsening with no obvious explanation. Given the high incidence of PE in critically ill COVID-19 patients, venous thromboprophylaxis with unfractionated or low-molecular-weight heparins appears of paramount importance. Increasing the prophylaxis towards high-prophylactic doses [17] with adjunctive leg compression may be discussed, even in absence of high level of evidence. Further larger studies are needed to assess the real prevalence and the risk factors of pulmonary embolism together with its prognostic impact on critically ill patients with COVID-19 ARDS.
